# Effect of ART scale-up and female migration intensity on risk of HIV acquisition: results from a population-based cohort in KwaZulu-Natal, South Africa

**DOI:** 10.1186/s12889-019-6494-x

**Published:** 2019-02-14

**Authors:** Armstrong Dzomba, Andrew Tomita, Alain Vandormael, Kaymarlin Govender, Frank Tanser

**Affiliations:** 1 Africa Health Research Institute (AHRI), KwaZulu-Natal, South Africa; 2 School of Nursing and Public Health, University of KwaZulu-Natal, Durban, South Africa; 3 KwaZulu-Natal Research Innovation and Sequencing Platform (KRISP), University of KwaZulu-Natal, Durban, South Africa; 4 Centre for Rural Health, School of Nursing and Public Health, University of KwaZulu-Natal, Durban, South Africa; 5 Health Economics and HIV and AIDS Research Division (HEARD), University of KwaZulu-Natal, Durban, South Africa; 6 Centre for the AIDS Programme of Research in South Africa (CAPRISA), University of KwaZulu-Natal, Durban, South Africa; 7 Research Department of Infection & Population Health, University College London, London, UK

**Keywords:** Risk of HIV acquisition, Female migrants, Migration intensity, ART scale-up, New HIV infections, Universal test and treat, South Africa

## Abstract

**Background:**

Despite increased antiretroviral therapy (ART) coverage, the incidence of HIV infection among women in rural South Africa remains high. While many socio-demographic and behavioral factors have been identified, the effect of female migration intensity on the risk of HIV acquisition before and after ART scale-up has not been evaluated in the country.

**Methods:**

We followed 13,315 female participants aged 15–49 who were HIV-uninfected at baseline and recorded their migration events between 2004 and 2015. Using a Cox proportional hazard model, we estimated the time to HIV acquisition among the women, adjusting for annual migration intensity (high: ≥2 events/year, moderate = 1 event/year, and low = 0 event/year) before and after ART scale-up in 2010.

**Results:**

1998 (15%) new HIV-infection events were recorded during the observation period. Overall, high migration intensity was associated with an increased HIV acquisition risk among women when compared with low migration intensity (HR = 2.88, 95% CI: 1.56–5.53). Among those with high migration intensity, the risk of HIV acquisition was significantly lower in the post-ART period compared to the pre-ART period, after controlling for key socio-demographic and behavioural covariates (aHR = 0.18, 95% CI 0.04–0.83).

**Conclusions:**

Women who migrated frequently after ART scale-up had a significantly reduced HIV acquisition risk compared to those before its implementation. While this reduction is encouraging, women who migrate frequently remain at high risk of HIV acquisition. In the era of ART, there remains a critical need for public health interventions to reduce the risk of HIV acquisition in this highly vulnerable population.

## Background

Human migration and mobility have played an important role in the spread of HIV in sub-Saharan African settings [1–3]. In South Africa, a country with the world’s highest and heaviest burden of HIV, the spatial, social and economic legacy of Apartheid and its racially-based migrant labour policies have continued to drive the AIDS epidemic since 1994 [4]. In recent years, the scale-up of ART in South Africa has been associated with substantial reductions in the risk of HIV acquisition [5–7]. However, the results from a recent mathematical model predict that high levels of migration in South African could attenuate the preventive benefits of ART over the next 30 years [8].

In recent years, several studies have shown that migration is associated with a higher risk of HIV acquisition [9–15], with a number of socio-demographic or behavioral factors explaining this relationship. For example, individuals who migrate or migrate frequently are less likely to engage with the local health-care system where they can access preventative HIV services [16, 17]. Increased levels of migration may also weaken the spousal bond through long absences away from home [9, 18–20], leading to greater opportunities for casual and transactional sexual contact. Despite an abundance of studies on migration and HIV acquisition risk [20, 21], most have focused on male migration [15, 17], with a few notable exceptions [22, 23]. Furthermore, there is lack of empirical evidence on whether the frequency of migration will attenuate the preventative benefits of ART, which is highly relevant in the era of universal access to HIV test and treat services.

The study aimed to quantify the effect of public sector ART scale-up in South Africa on the HIV acquisition risk among migrant women by comparing pre- and post-implementation data from one of the world’s largest population-based HIV testing platforms that is maintained by the Africa Health Research Institute (AHRI).

## Methods

### Study area

The study was conducted in the rural AHRI demographic and health surveillance area in the uMkhanyakude District of KwaZulu-Natal Province, South Africa. The surveillance area is home to approximately 90,000 resident and non-resident participants from 11,000 households [24]. Data are collected from all households within the study area. The eligibility criteria for inclusion of individuals in these households is based on membership status, with members being either resident or non-resident, i.e. residing at the same homestead at a point in time or living elsewhere but maintaining ties to households in the study area. The AHRI surveillance and HIV cohorts are dynamic open cohorts in which, on an ongoing basis, individuals leave and enter each cohort via outmigration or death and in-migration or birth respectively. Participation rates for household data collection within the surveillance system are high at > 95% on each wave [24]. To ensure collection of complete and accurate data the household head or the most senior household member is identified as the key informant and best person to provide information. If unavailable other suitable household members are selected as alternative informants. The study assumed that the key informant is cognizant about the migration and resident status of household members. HIV incidence is high, with approximately 2.6 infections per 100 person-years (95% CI 2.5–2.8) over the 2004–2010 period. In 2007, the incidence peaked at 6.6 infections per 100 person-years among women aged 24 years in 2007 [5], and among migrants, the prevalence was highest in women aged 25–29 years at 63% (95% CI 50–76%) and at 56% (95% CI 34–78%) among men aged 35–39 years [25]. ART was introduced into the 17 primary health-care (PHC) clinics in and adjacent to the surveillance area in 2004, with a CD4 count eligibility of < 200 cells per μL. In 2010, treatment eligibility was extended to persons with CD4+ T-cell counts < 350 cells/μL, pregnant woman, and patients with active tuberculosis. ART has been freely available to all HIV-infected persons since 2016, in line with the World Health Organization (WHO) guidelines.

### Study design and data collection

The current study utilized prospective population-based cohort data collected from the AHRI surveillance area between 2004 and 2015. Records for key demographic events, such as births, deaths and migrations, were collected by trained field-workers from key household informants every 4–6 months. The socio-economic and health measures were collected annually, and individual HIV testing took place yearly since 2003. Eligible participants aged 15-years and older are interviewed in private by the same fieldworkers, who also extract blood from consenting participants by finger-prick for HIV testing. The Biomedical Research Ethics Committee of the University of KwaZulu-Natal (BREC) Durban, South Africa, gave full ethics approval for this study.

### Measurement

#### HIV outcome

The primary outcome of the study was the risk of HIV acquisition among women. All female participants with two or more valid HIV test results, for whom the first HIV test was negative, were included in the incident cohort, which is consistent with previous studies [10, 26, 27]. As the seroconversion date is known only to occur between the latest HIV-negative and earliest HIV-positive test date, we imputed the seroconversion date [28]. We right censored the data at the imputed seroconversion date, or at the latest HIV-negative date, if no HIV-positive test result was observed during the study period.

#### Migration intensity

The AHRI surveillance system records several measures of migration, including dates of in- and out-migration from the study area and the place of destination. In the current study, we defined migration as a movement out of the surveillance area (i.e. external migration) at least once during the observation period. On average, the annual number of migration events per individual can range from 0 to 3 and the duration of external migration from the surveillance area must be at least 4 months. Given that the migration history was provided by the key household informant, participation rates for data collection within the AHRI surveillance system are > 95% [24]. We measured migration intensity as the total number of external migration events per year, categorized as: high: ≥2 events/year, moderate = 1 event/year, and low = 0 event/year.

#### ART scale-up

We defined two ART periods: pre-ART scale-up (2004–2009) and post-ART scale-up (2010–2015), in line with previous analyses from the surveillance area [29, 30].

### Statistical analysis

We provide descriptive statistics of the key socio-demographic and behavioural variables, including: sex, age, marital status, pregnancy history, contraception use, sexual debut age, number of lifetime sexual partners, household wealth and area of residence. Information on contraception is based on questions related to ever engaging in birth control practices, such as using contraceptive pills, condoms and injectable methods. Three overarching analyses were conducted. First, we examined the abovementioned factors of migration intensity using bivariate and multivariate ordered logistic regression models. The ordered logistic regression models accounted for repeated measures, meaning that the annual number of migration events could vary over time for each participant. To account for clustering of observations within each individual, we estimated robust SEs in the analysis (Table 4). Second, HIV incidence rates were calculated per the abovementioned socio-demographic and behavioural factors. Third, we fitted Cox proportional hazard models to estimate the effect of ART scale-up period on the HIV acquisition risk by controlling for migration intensity and the socio-demographic and behavioural factors. In the multivariate model, we included an interaction term between ART scale-up period and migration intensity. We undertook a post-estimation analysis to compare the hazard of HIV acquisition across the pre- and post-ART period, holding migration intensity and other socio-demographic and behavioral factors constant. All analyses were undertaken in Stata 15 (StataCorp, College Station, Texas, USA), with the *lincom* command being used for the post-estimation analyses.

## Results

### Baseline demographic characteristics

A total of 13,315 female participants aged 15 to 49 years at baseline were followed-up, their demographic characteristics being listed in Table 1. Of the female participants, 41% were aged between 15 and 24 years and approximately a third (35%) had experienced sex by the age of 17 years (results not shown).

**Table 1:** Baseline socio-demographic characteristics of women in the AHRI population-based HIV incidence cohort (*N* = 13,315)

		n	%
Age category:	15–19	2555	19.19
	20–24	2828	21.24
	25–29	1290	9.69
	30–34	667	5.01
	35–39	496	3.73
	40+	5479	41.15
Marital status:	Single	9040	69.38
	Divorced/Separated	2031	15.59
	Married - Monogamous	1745	13.39
	Married - Polygamous	214	1.64
Sexual debut	After the age of 17	2782	34.97
	After 17	5173	63.03
Household income:	Bottom 20%	2535	20.25
	Middle 60%	7633	60.96
	Top 20%	2353	18.79
Ever pregnant	Yes	10,486	83.41
	No	2086	16.59
Contraceptive use	Yes	2830	81.02
	No	663	18.98
Number of sex partners	Less than 5 partners	9934	98.38
	≥5	164	1.62
Area:	Peri-urban	454	3.59
	Urban	3591	28.38
	Rural	8610	68.04
Number of external migration movements per year:	None	12,357	93.15
	One movement per year	777	5.86
	Two or more movements per year	132	1.00

Note: Due to missing data, values in certain category does not add up to *N* = 13,315

### Predictors of external migration

Table 2 shows the bivariate and multivariate ordinal logistic regression results for the predictors of external migration events. Several socio-demographic and behavioural factors were predictive of external migration. Increased odds of migration was associated with being aged 20–24 years (aOR = 4.46, 95% CI 3.11–6.39) compared to 40 years and above, having no history of pregnancy (aOR = 1.50, 95% CI 1.08–2.06) compared to ever pregnant, having sexual debut after the age of 17 (aOR = 1.22, 95% CI 1.02–1.45) compared to before age 17, residence in urban areas (aOR = 1.72, 95% CI 1.15–2.55) compared to rural areas, and living in households in the top 20% of relative wealth (aOR = 1.28, 95% CI 1.03–1.58) compared to the middle 60% of relative wealth.

**Table 2:** Predictors of external migration movements based on ordered logistic regression models

	Category	OR	aOR	Robust SE	95% CI	
Age category: [40+]	15–19	3.85***	2.36***	0.48	1.58	3.51
	20–24	9.84***	4.46***	0.82	3.11	6.39
	25–29	7.91***	2.96***	0.59	1.99	4.38
	30–34	4.25***	1.57	0.37	0.99	2.49
	35–39	3.13***	1.44	0.35	0.90	2.32
Marital status: [Single]	Divorced/Separated	0.12***	0.24	0.18	0.06	1.00
	Married - Monogamous	0.15***	0.43***	0.09	0.28	0.65
	Married - Polygamous	0.07***	0.15	0.15	0.02	1.12
Sexual debut age: [Before age of 17]	After 17	1.01	1.22***	0.11	1.02	1.45
Ever pregnant: [Yes]	No	0.92	1.50***	0.25	1.08	2.06
Contraception use: [Yes]	No	2.26***	1.19	0.12	0.98	1.45
Number of sexual partners: [Less than 5 partners]	≥5	1.47*	0.62	0.19	0.34	1.13
Household income: [Middle 60%]	Bottom 20%	0.95	1.15	0.12	0.94	1.42
	Top 20%	1.03	1.28***	0.14	1.03	1.58
Area: [Rural]	Peri-urban	0.88***	0.92	0.09	0.76	1.12
	Urban	1.29**	1.72	0.35	1.15	2.55
ART scale-up period: [Year 2010 and on]	Before 2010	1.63***	1.19	0.11	0.99	1.43

* *p* < 0.05, **p < 0.01, ***p < 0.001

Reference category in bracket

The above final regression result is based on *n* = 5978

### Incidence rates

The crude HIV incidence rate was 3.16 infections per 100 person-years over the 63,217 person-years of follow-up between 2003 and 2015. Table 3 shows that the crude incidence rate for high intensity of migration (two or more times per year) was three times higher (8.56 per 100 person-years) compared to those with low migration intensity (no migration events) (2.97 per 100 person-years). Overall, there was a slight decline in HIV incidence in the post-ART period (2.83 per 100 person-years) compared to the pre-ART period (3.53 per 100 person-years).

**Table 3:** HIV incidence rates among women who migrate in the AHRI population-based HIV incidence cohort 2003–2015, by socio-demographic factors

		Events	Person years	IR (per 100 person years)
Age category:	15–19	529	11,085	4.77
	20–24	734	9876	7.43
	25–29	321	4719	6.80
	30–34	128	3088	4.14
	35–39	67	3430	1.95
	40+	219	31,018	0.71
Marital status:	Single	1733	38,457	4.51
	Divorced/Separated	36	10,624	0.34
	Married - Monogamous	108	12,095	0.89
	Married - Polygamous	10	1640	0.61
Sexual debut age:	Before the age of 17	590	11,139	5.30
	Ages 17 and after	750	22,164	3.38
Ever pregnant:	No	143	4368	3.27
	Yes	1332	44,794	2.97
Contraception use:	No	209	4941	4.23
	Yes	478	8114	5.89
Number of sexual partners:	Less than 5 partners	1737	51,428	3.38
	≥5	57	850	6.70
Household income:	Bottom 20%	329	10,664	3.09
	Middle 60%	1166	35,543	3.28
	Top 20%	272	10,243	2.66
Area:	Peri-urban	624	16,625	3.75
	Urban	54	1738	3.11
	Rural	1213	42,835	2.83
Number of external migration movements per year:	None	1780	59,943	2.97
	One movement per year	168	2677	6.27
	Two or more movements per year	48	561	8.56
ART scale-up period:	Before 2010	953	33,638	3.53
	Year 2010 and on	1045	29,579	2.83

The above final regression result is based on *n* = 5978

### Cox proportional hazard results

Of the 13,315 participants, 1998 (15%) had an HIV-infection event. The bivariate analyses in Table 4 (column 1) show that, compared with the pre-ART period, the risk of HIV acquisition among women declined significantly in the post-ART period (HR: 0.81, 95% CI: 0.73–0.90). After controlling for socio-demographic and behavioral covariates, the risk of HIV acquisition among women with high migration intensity declined significantly in the post-ART period compared with high migration intensity in the pre-ART period (*lincom* results: aHR = 0.19, 95% CI: 0.04–0.87). A lower risk of HIV acquisition was associated with marital status compared to being single (aHR = 0.48, 95% CI: 0.31–0.77), and with having sexual debut after the age of 17 years compared to before (aHR = 0.83, 95% CI: 0.69–0.98), as shown in Column 2 of Table 4.

**Table 4:** The relationship between female migration and risk of HIV acquisition before and after ART scale-up period in the AHRI population-based HIV incidence cohort

	Category	HR	aHR	SE	95% CI	
Age category: [40+]	15–19	7.39***	4.98***	0.63	3.14	7.89
	20–24	10.86***	5.61***	0.85	3.63	8.68
	25–29	9.46***	4.15***	0.83	2.63	6.56
	30–34	5.83***	2.51***	0.65	1.51	4.15
	35–39	2.80***	1.46	0.39	0.83	2.56
Marital status: [Single]	Divorced/Separated	0.07***	0.97	0.51	0.35	2.74
	Married - Monogamous	0.19***	0.48***	0.11	0.31	0.77
	Married - Polygamous	0.13***	0.27	0.28	0.04	1.97
Sexual debut age: [Before age of 17]	After 17	0.64***	0.83***	0.07	0.69	0.98
Ever pregnant: [Yes]	No	1.00	1.20	0.24	0.81	1.77
Contraception use: [Yes]	No	0.72***	1.00	0.11	0.81	1.24
Number of sexual partners: [Less than 5 partners]	≥5	1.99***	0.75	0.21	0.44	1.29
Household income: [Middle 60%]	Bottom 20%	0.93	1.07	0.12	0.86	1.34
	Top 20%	0.80***	0.91	0.11	0.71	1.16
Area: [Rural]	Peri-urban	1.38***	1.32***	0.12	1.10	1.58
	Urban	1.22	0.84	0.29	0.43	1.64
Number of external migration movements per year: [None]	One movement per year	2.10***	0.90	0.23	0.54	1.49
	Two or more years movement per year	2.88***	2.94***	0.94	1.56	5.53
ART scale-up period: [Before 2010]	Year 2010 and on	0.81***	1.04	0.12	0.82	1.32
Interaction terms of annual movement and ART-scale-up period [Before 2010]	Once per-year/2010 and on		1.21	0.50	0.53	2.72
	Twice or more per-year/2010 and on		0.18***	0.14	0.04	0.83

* *p* < 0.05, **p < 0.01, ***p < 0.001 only for bivariate analyses

Reference category in bracket

## Discussion

Our study in a rural South African community found that among women who had high annual migration intensity, the risk of HIV acquisition was lower in the post-ART period (2010–2015) than in the pre-ART period (2004–2009). Nevertheless, the risk of HIV acquisition was considerably higher among women who migrated compared to those who did not.

Our findings are comparable to a similar study from Rakai, Uganda, where the HIV incidence rate ratio in recent female migrants during the 2011–2015 period was lower than in those who migrated between 2004 and 2011 (which corresponds with our pre-ART period), although this difference was not statistically significant [15]. Despite the difference in the definition of migration (i.e. migration recency vs. migration intensity), we found that high intensity migrants (≥2 events per year) had a significantly lower risk of HIV acquisition in the post-ART than in the pre-ART period, after controlling for socio-demographic and behavioral covariates. We posit that increased ART scale-up had improved treatment coverage among their sexual partners and access to preventative HIV services (e.g. availability of condom and education) among the women and their sexual partners in the post ART scale-up period, leading to overall lower HIV acquisition risk.

There are several limitations for our study. First, HIV testing rates may have been different across the pre- and post-ART scale-up periods. To assess this possibility, we looked at the average amount of time between the latest HIV-negative and earliest HIV-positive test dates by ART scale-up period. We found that there was a significant difference in the average censored interval lengths, which was 2.87 years in the pre-ART versus 3.19 years in the post-ART period (*p* = 0.002). Second, the sexual behaviour covariates measures (e.g. number of sexual partners and sexual debut age) were based on self-reports. We acknowledged that the low response rate (as indicated in Table 1), possibly due to social desirability biases, may have impacted the representativeness of our result. We acknowledge here that attrition is a challenge in the AHRI surveillance area. The issue of attrition has been closely investigated by Larmarange and colleagues. During five-year period in the study area, over two-thirds participated at least once, 48% at least twice and 31% at least three times [31], raising the possibility of about biases in incidence estimation.

Notwithstanding these limitations, the strengths of our study include the follow-up of more than 13,000 women participating in one of the worlds’ largest and longest running HIV-focused cohorts, and enabled us to capture long-term trends in female HIV acquisition risk. We also collected comprehensive data on the migration history of the female participants for more than a decade, which allowed us to measure the impact of long-term time-varying migration frequency on the risk of HIV acquisition.

Our study findings have highlighted the need to identify frequent migrants and rapidly link them to HIV treatment and prevention services. Women who migrate are particularly vulnerable to HIV and may benefit from accessing pre-exposure prophylaxis (PrEP), only offered to commercial sex workers [32] at the time of this study. Women with high levels of migration intensity may also benefit from mobile technology scale-up (i.e. *mHealth,* text message-based applications). Such technology can improve the ability of the healthcare system to track and interact with migrants as they move from one place to another, ensuring better health outcomes and healthcare delivery as well as continued HIV surveillance [33]. Additionally, there is also empirical evidence for the efficacy of mobile community health workers to achieve HIV treatment and prevention [34]. This strategy involves scaling-up home-based testing to complement universal counselling and testing currently accompanying ART services [35], and may be beneficial if such interventions target the homes of frequent female migrants.

## Conclusions

Our study found that among women with high annual migration intensity, the risk of HIV acquisition was significantly lower in the post-ART period than the pre-ART scale-up period. Nevertheless, women who migrate frequently remain at a higher risk of HIV acquisition. Innovative primary prevention programs focused on females who frequently migrate are urgently needed to reduce the risk of HIV acquisition among this highly vulnerable group.

## Abbreviations

AHRI: Africa Health Research Institute
ART: Antiretroviral Therapy
BREC: Biomedical Research Ethics Committee
HIV: Human Immune Virus
KZN: KwaZulu-Natal
PrEP: Pre-Exposure Prophylaxis

## References

[1] Lurie MN, Williams BG, Zuma K, Mkaya-Mwambury D, Abdool Karim SS (2003). The impact of migration on HIV-1 transmission in South Africa: a study of migrants and non-migrant men and their partners. Sex Transm Dis.

[2] Voeten HACM, Vissers DCJ, Gregson S, Zaba B, White RG, de Vlas SJ (2010). Strong association between in-migration and HIV prevalence in urban sub-Saharan Africa. Sex Transm Dis.

[3] Cassels S, Jenness SM, Biney AAE, Ampofo WK, FN-A D (2014). Migration, sexual networks, and HIV in Agbogbloshie, Ghana. Demogr Res.

[4] Lurie MN, Williams BG (2014). Migration and health in southern Africa: 100 years and still circulating. Heal Psychol Behav Med.

[5] Vandormael A, Newell M-L, Bärnighausen T, Tanser F (2014). Use of antiretroviral therapy in households and risk of HIV acquisition in rural KwaZulu-Natal, South Africa, 2004–12: a prospective cohort study. Lancet Glob Health.

[6] Tanser F, Baernighausen T, Graspa E, Zaidi J, Newell M-L (2013). High coverage of ART associated with decline in risk of HIV acquisition in rural KwaZulu-Natal. Science.

[7] Oldenburg CE, Bor J, Harling G, Tanser F, Mutevedzi T, Shahmanesh M, Seage GR, De Gruttola V, Mimiaga MJ, Mayer KH, Pillay D. Impact of early antiretroviral therapy eligibility on HIV acquisition: household-level evidence from rural South Africa. AIDS 2018;32(5):635. 10.1097/QAD.0000000000001737 PMC5832606 29334546

[8] Andrews JR, Wood R, Bekker L-G, Middelkoop K, Walensky RP (2012). Projecting the benefits of antiretroviral therapy for HIV prevention: the impact of population mobility and linkage to care. J Infect Dis.

[9] McGrath N, Eaton JW, Newell M-L, Hosegood V (2015). Migration, sexual behaviour, and HIV risk: a general population cohort in rural South Africa. Lancet HIV.

[10] Bärnighausen T, Hosegood V, Timaeus IM, Newell M-L (2007). The socioeconomic determinants of HIV incidence: evidence from a longitudinal, population-based study in rural South Africa. AIDS.

[11] Townsend L, Giorgio M, Zembe Y, Cheyip M (2014). HIV prevalence and risk behaviours among foreign migrant women residing in Cape Town, South Africa. AIDS Behav.

[12] Hargreaves JR, Bonell CP, Morison LA, Kim JC, Phetla G, Porter JD (2007). Explaining continued high HIV prevalence in South Africa: socioeconomic factors, HIV incidence and sexual behaviour change among a rural cohort, 2001–2004. AIDS.

[13] Dobra A, Bärnighausen T, Vandormael A, Tanser F (2017). Space-time migration patterns and risk of HIV acquisition in rural South Africa. AIDS.

[14] Dzomba A, Tomita A, Govender K, Tanser F. Effects of migration on risky sexual behavior and HIV acquisition in South Africa: a systematic review and meta-analysis, 2000-2017. AIDS Behav. 2018:1–35. 10.1007/s10461-018-2367-z 30547333

[15] Olawore O, Tobian AAR, Kagaayi J, Bazaale JM, Nantume B, Kigozi G (2018). Migration and risk of HIV acquisition in Rakai, Uganda: a population-based cohort study. Lancet HIV..

[16] Phillips TK, Clouse K, Zerbe A, Orrell C, Abrams EJ, Myer L (2018). Linkage to care, mobility and retention of HIV-positive postpartum women in antiretroviral therapy services in South Africa. J Int AIDS Soc.

[17] Clouse K, Fox MP, Mongwenyana C, Motlhatlhedi M, Buthelezi S, Bokaba D (2018). “I will leave the baby with my mother”: long-distance travel and follow-up care among HIV-positive pregnant and postpartum women in South Africa. J Int AIDS Soc.

[18] Platt L, Grenfell P, Fletcher A, Sorhaindo A, Jolley E, Rhodes T, Bonell C (2013). Systematic review examining differences in HIV, sexually transmitted infections and health-related harms between migrant and non-migrant female sex workers. Sex Transm Infect.

[19] Saggurti N, Nair S, Malviya A, Decker MR, Silverman JG, Raj A (2012). Male migration/mobility and HIV among married couples: cross-sectional analysis of nationally representative data from India. AIDS Behav.

[20] Camlin CS, Akullian A, Neilands TB, Getahun M, Eyul P, Maeri I (2018). Population mobility associated with higher risk sexual behaviour in eastern African communities participating in a universal testing and treatment trial. J Int AIDS Soc.

[21] Schuyler AC, Edelstein ZR, Mathur S, Sekasanvu J, Nalugoda F, Gray R (2015). Mobility among youth in Rakai, Uganda: trends, characteristics, and associations with behavioural risk factors for HIV. Glob Public Heal.

[22] Posel D (2010). Households and labour migration in post-apartheid South Africa. J Stud Econ Econometrics.

[23] Camlin CS, Snow RC, Hosegood V (2014). Gendered patterns of migration in rural South Africa. Popul Space Place.

[24] Tanser F, Hosegood V, Barnighausen T, Herbst K, Nyirenda M, Muhwava W (2008). Cohort profile: Africa Centre demographic information system (ACDIS) and population-based HIV survey. Int J Epidemiol.

[25] Welz T, Hosegood V, Jaffar S, Bätzing-Feigenbaum J, Herbst K, Newell ML (2007). Continued very high prevalence of HIV infection in rural KwaZulu-Natal, South Africa: a population-based longitudinal study. AIDS.

[26] Tomita A, Vandormael AM, Barnighausen T, de Oliveira T, Tanser F (2017). Social disequilibrium and the risk of HIV acquisition. J Acquir Immune Defic Syndr.

[27] Tanser F, Vandormael A, Cuadros D, Phillips AN, de Oliveira T, Tomita A, Bärnighausen T, Pillay D (2017). Effect of population viral load on prospective HIV incidence in a hyperendemic rural African community. Sci Transl Med.

[28] Vandormael A, Dobra A, Bärnighausen T, de Oliveira T, Tanser F (2018). Incidence rate estimation, periodic testing and the limitations of the mid-point imputation approach. Int J Epidemiol.

[29] Hontelez JAC, Tanser FC, Naidu KK, Pillay D, Bärnighausen T, Saltzman A (2016). The effect of antiretroviral treatment on health care utilization in rural South Africa: a population-based cohort study. PLoS One.

[30] Zaidi J, Grapsa E, Tanser F, Newell M-L, Bärnighausen T (2013). Dramatic increase in HIV prevalence after scale-up of antiretroviral treatment. AIDS.

[31] Larmarange J, Mossong J, Bärnighausen T, Newell ML (2015). Participation dynamics in population-based longitudinal HIV surveillance in rural South Africa. PLoS One.

[32] Kharsany ABM, Karim QA (2016). HIV infection and AIDS in sub-Saharan Africa: current status, challenges and opportunities. Open AIDS J.

[33] Tanser F, Bärnighausen T, Vandormael A, Dobra A (2015). HIV treatment cascade in migrants and mobile populations. Curr Opin HIV AIDS.

[34] Bärnighausen T, Chaiyachati K, Chimbindi N, Peoples A, Haberer J, Newell ML (2011). Interventions to increase antiretroviral adherence in sub-Saharan Africa: A systematic review of evaluation studies. Lancet Infect.

[35] Chamie G, Clark TD, Kabami J, Kadede K, Ssemmondo E, Steinfeld R, Lavoy G, Kwarisiima D, Sang N, Jain V, Thirumurthy H (2016). A hybrid mobile approach for population-wide HIV testing in rural East Africa: an observational study. Lancet HIV..

